# Mental health hospitalization and readmission in autistic adults in a national U.S. sample

**DOI:** 10.1016/j.reia.2025.202582

**Published:** 2025-04-02

**Authors:** Jessica E Rast, Joseph Wright, Samuelle Voltaire, Tamara Garfield, Anne M Roux

**Affiliations:** A.J. Drexel Autism Institute, Drexel University, Philadelphia, PA, USA

**Keywords:** Mental health hospitalization, Readmission, Autism, Adults

## Abstract

**Objective:**

The objectives of this study were to, 1) estimate U.S. national rates of mental health hospitalization (MHH) and all-cause readmission, and 2) explore characteristics associated with readmissions for autistic and non-autistic adults.

**Methods:**

This study used the National Readmission Database (NRD) 2019 to examine 30-day all-cause readmissions following MHH in adults with autism, intellectual disability, ADHD, and mental health conditions. Analysis estimated rates of MHH by group, rates and odds of readmission, and factors associated with readmission.

**Results:**

More than one-third (36 %) of all admissions for autistic adults in 2019 were MHH, with schizophrenia the most common reason. Of all MHH index events in autistic adults, 17 % had a 30-day all-cause readmission. Readmissions were more common in autistic adults with ID (25 %) than in autistic adults without ID (15 %). Readmission was also slightly more common in female autistic adults (20 %) than male autistic adults (16 %), and autistic females had greater odds of readmission (OR 1.30, 95 % CI 1.09, 1.55) than autistic males, which was not true in non-autistic adults. Initial admission for schizophrenia was associated with increased odds of readmission for all groups (OR 1.37, 95 % CI 1.32, 1.42).

**Conclusions:**

The complexity of mental health conditions and their care in autistic adults underscores the urgent need for accessible and tailored mental health care services. Addressing these challenges will require collaborative efforts across healthcare sectors to create comprehensive, inclusive, and person-centered approaches to mental health care delivery for autistic adults across often disjointed service sectors.

## Introduction

Mental health conditions are common among autistic adults; half have a co-occurring condition, with anxiety, depression, and ADHD the most common (Lugo-Marin et al., 2019; Schott et al., 2021). Co-occurring mental health conditions are more common among autistic adults than their non-autistic peers, adversely impacting the quality of life, complexity, and cost of health care for autistic adults, creating a complex clinical picture (Croen et al., 2015; Lai et al., 2019; Rast et al., 2021; Zerbo et al., 2019). High rates of mental health conditions in autistic adults may be partially attributed to a service system that lacks an evidence base for effective care, stigma, trauma and financial hardship accumulating across the life course, and a desire for, but lack of, interpersonal social support (Dodds, 2021; Hedley et al., 2017; Hull et al., 2021; Maddox et al., 2020; Mitchell et al., 2021). Some studies also suggest there may be diagnostic overlap or shared etiology with autism and certain conditions, such as schizophrenia (King & Lord, 2011; Zheng et al., 2018). Mental health conditions, including mood disorders and schizophrenia, are the two most common reasons for inpatient hospitalization in autistic adults (Rast et al., 2021).

Mental health services play a key role in supporting people with mental health diagnoses by supporting symptom management for community participation (Maddox et al., 2020; Maddox & Gaus, 2019). Accessible services prevent an increase in the severity of mental health symptoms and decrease risk of crisis events (Maddox et al., 2020; Maddox & Gaus, 2019). However, the lack of an integrated mental health system across primary and specialty care results in gaps in care that lead to mental health crises and use of emergency and inpatient hospitalization as a recurrent care modality (Burns, 2017). These settings are not ideal treatment locations, as they often lack resources, training in autism competent care, and linkages to long term treatment options; care delivered in these settings is often more costly, intensive, and intrusive than community-based preventive care (Drake & Bond, 2021; Krupski et al., 2016; Navas et al., 2022). If hospitalization does occur, appropriate treatment and discharge instructions can connect a person to outpatient mental health care to reduce the risk of recurring crisis care.

Very little is known about readmission risk and associated factors in autistic adults. In non-autistic populations, previous studies have identified predictors of readmission after a mental health hospitalization (MHH) including diagnoses of substance use disorders, psychosis, and major depression; longer length of hospital stay; hospitalization and service use prior to MHH (Becker et al., 2017; Cheng et al., 2017; Kartha et al., 2007; Perlman et al., 2015); demographics and social determinants of health including race, sex, urbanicity, household income (Carroll et al., 2022); and social belonging (Zhao et al., 2020). People who do not receive appropriate follow-up care are more likely to experience hospital readmissions, service and medication non-compliance, and self-injurious and dangerous behaviors (Choi et al., 2020; Greenberg & Rosenheck, 2005; Puntis et al., 2015). People who are readmitted within 30 days experience an increased risk of two year all-cause mortality (Shaw et al., 2020). In a study of autistic youth, one of the few on readmission within autism, specific antipsychotic medication initiation following MHH was associated with reduced risk of psychiatric re-admission (Kompella et al., 2022). Readmission within 30 days is a common measure of health care quality; for example, it is the measure of readmissions used by the Hospital Readmissions Reduction Program by the Centers for Medicare and Medicaid Services (CMS).

Autistic adults have unique presentation and support needs that suggest their readmission risks may be different from non-autistic adults, requiring targeted inquiry. The accessibility of appropriate mental health care is limited in autistic adults by provider availability, impacting the ability to connect to outpatient care after discharge (Maddox et al., 2020). Even if there were providers to discharge to, hospital staff may not have resources or training in autism to deploy a thorough and accessible discharge plan. This planning is made more complex because many state policies and programs were designed with autistic children, not adults, in mind, and therefore autistic adults may not qualify for community services (Turcotte et al., 2016). The common co-occurrence of certain conditions in autistic adults, including intellectual disability (ID) and attention-deficit/hyperactivity disorder (ADHD), also complicates mental health care. These conditions commonly co-occur with autism and are themselves complicating conditions for mental health care. Patients without autism who have ID or ADHD may have similar mental health profiles and increased service needs as autistic adults, making them a useful comparison group for study.

The purpose of this study is to describe metal health hospitalizations (MHH) and all-cause readmission in autistic adults to begin to understand the specific needs of this population. Many studies of mental health readmission are international or are from a geographically limited area if conducted in the U.S., potentially due to the accessibility of data that allows the examination of readmission (Becker et al., 2017; Carroll et al., 2022; Cheng et al., 2017; Kartha et al., 2007; Perlman et al., 2015). To address this limitation, this study uses the National Readmission Database (NRD), which is a nationally representative sample of hospital discharges built to study readmissions. NRD allows for an all-payer, nationally representative examination of hospital admissions and readmissions within a calendar year. Using this data, we aim to 1) quantify national MHH characteristics and all-cause readmission and 2) examine characteristics associated with readmission for autistic and non-autistic adults.

## Methods

### Data source and population

This study used the National Readmission Database (NRD) 2019, a research database cataloging hospitalizations over a calendar year with the intent of performing readmission analysis. NRD is created from State Inpatient Databases by the Healthcare Cost and Utilization Project (HCUP) of Agency for Healthcare Research and Quality (AHRQ). Hospitalizations captured in NRD are all-payer admissions to community hospitals, excluding rehabilitation and long-term acute care hospitals. All admissions in a given year are linked via a personal identifier. Analysis of NRD is nationally representative; the 2019 data is comprised of about 18 million discharges (weighted to 35 million discharges) from 30 states with reliable patient linkage.

### Index stay and all-cause readmission

Index stays were stays for a mental health hospitalization (MHH) captured as stays with any Clinical Classifications Software Refined (CCSR) code starting with mental behavioral disorder (“MBD”) in the primary position of the stay record, excluding autism, intellectual disability (ID), and attention deficit hyperactivity disorder (ADHD) based on the below-described ICD-10 codes. The primary position of the stay record, also called the principal diagnosis, is the first ICD code listed in the discharge record (of a possible 40 code positions). The principal diagnosis is generally considered to be the primary reason for a hospitalization, with some variability in coding based on insurance requirements and clinical judgement (McDermott KW (IBM Watson Health) & Roemer M (AHRQ), 2021). CCSR is a classification system that collapses international classification of diseases (ICD) codes into fewer, more clinically meaningful codes for research. Index events were limited to exclude hospitalizations beginning in January or December, to ensure that there were 30 days of observation before and after the index event to capture prior admissions or readmissions. An index event must have been the first mental health hospitalization within a 30-day period, to ensure they were not themselves a readmission.

This study used 30-day all-cause readmission as the outcome of interest. This was captured as any hospital admission within 30 days of discharge for the index event. All-cause readmission is a consistent care quality indicator and points to the accessibility of outpatient care after discharge.

### Populations

This study compared four mutually exclusive groups of adults ages 18 and older experiencing mental health hospitalizations: autistic adults, adults with ID, adults with ADHD, and adults with any other mental health condition. Mental health hospitalization was identified by the diagnosis in the primary position of the stay record. Therefore, populations were categorized by conditions in any secondary position of the stay record (any of position 2 through 40, the maximum number). Autistic adults were those with any ICD-10 code F84. X in a secondary position, they may also have had any of the other conditions of ID, ADHD, or another mental health condition. Adults with ID were adults with any ICD-19 code F70. X-F79. X in a secondary position, with no autism code in a secondary position but could have had an ADHD or other mental health diagnosis. Adults with ADHD were adults with an ICD-10 code F90. X in a secondary position, with no autism nor ID code in a secondary position. Adults with other mental health conditions were those with any CCSR code beginning with “MBD” in a secondary position, but no autism, ID, or ADHD codes in a secondary position. These groups were chosen due to an assumed similar underlying risk for mental health hospitalization (MHH), which assisted in examination of differences in services related specifically to autism.

### Patient and stay characteristics

Patient characteristics included age (grouped as 18–44, 45–64, and 65 years and older), sex, urbanicity, and the median household income of the patient’s zip code. Stay characteristics included length of stay, cost of stay, emergency department use before or during the admission, elective admission, primary expected payer, and presence of an injury code in the stay record. Specific mental health diagnoses that have been shown in prior research to be associated with greater risk of hospital readmission included substance use, depression, and schizophrenia in the principal position of the stay record (Becker et al., 2017; Kartha et al., 2007). Information on race and ethnicity was not available.

### Statistical analysis

We first presented the proportion of all hospitalizations for each group that were for mental health conditions to examine the magnitude of the MHH frequency in hospitalizations for each population. We then presented the distribution of characteristics of the index MHHs for each group, including the reason (the principal diagnosis) for the admission. For each group, we examined the 30-day all-cause readmission rate. We also examined 30-day all-cause readmission among autistic adults by several key groups: ID status, sex, and age group. No statistical significance testing was performed between groups in this portion of the study. We generally deemed a finding clinically significant if the difference between groups was greater than 5 %.

Among those who had a readmission, we identified the most common reasons for readmission by group. We then built two multivariable logistic regression models to examine: 1) the association of covariables with readmission, with group status as covariables, and 2) the association of covariables with readmission within the autistic group only, including ID status as a covariable. All analysis accounted for the complex sampling design of NRD and was performed using SAS 9.4.

## Results

### Mental health hospitalization

**Fig. 1.**
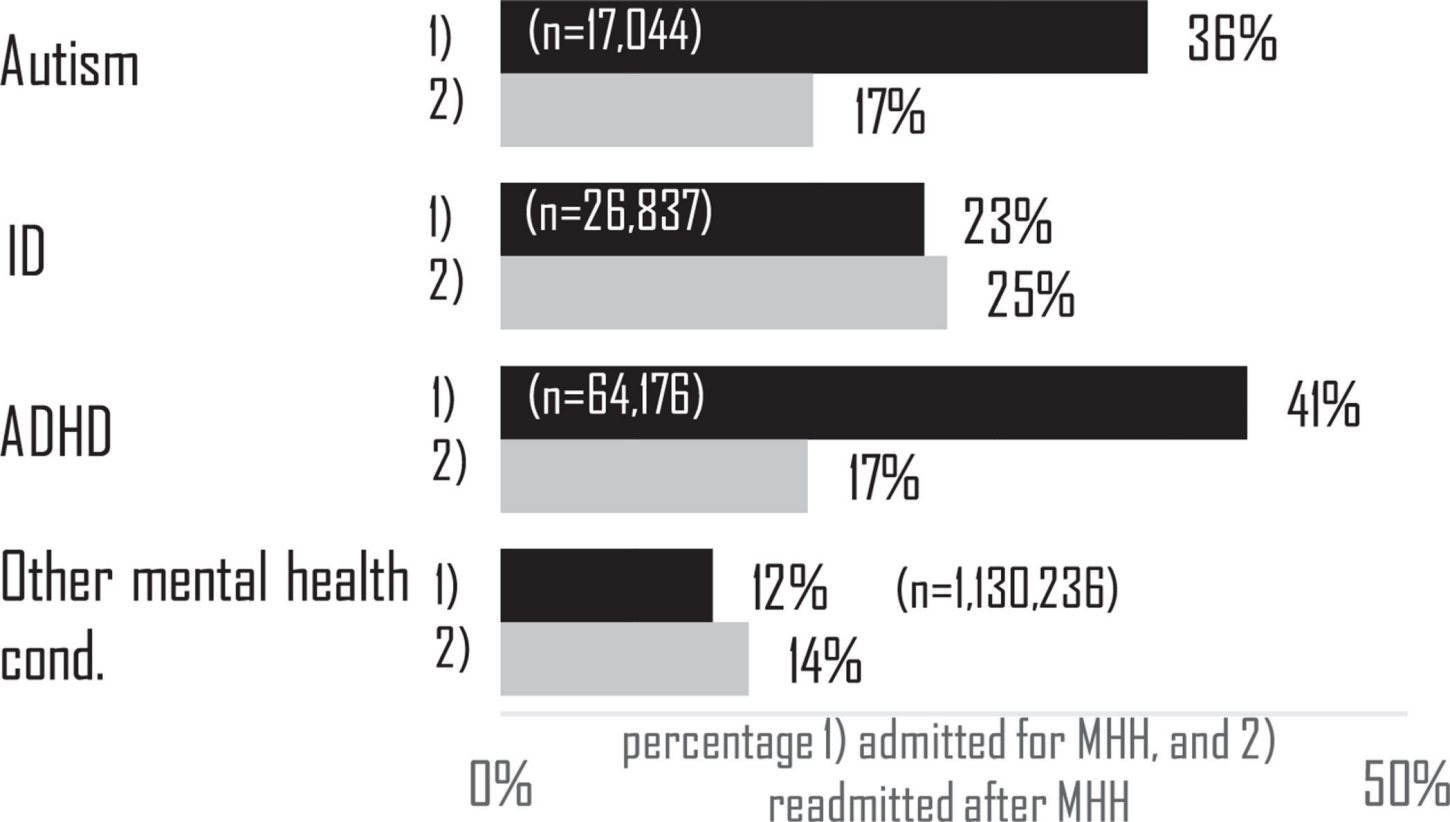
Prevalence of 1) mental health hospitalzation (weighted number of index events) and 2) 30-day readmission.

More than one-third (36 %) of all admissions for autistic adults in 2019 were for mental health hospitalizations (MHH), compared to 23 % for adults with ID, 41 % for adults with ADHD, and 12 % for adults with other mental health conditions. There were 17,044 MHH in 2019 that counted as an index event for autistic adults in this study (Fig. 1).

The patient and stay characteristics of the index MHH are seen in Table 1. Autistic adults were younger (average age 28.5), less often female (28.7 %), and more often lived in zip codes with higher median incomes than non-autistic adults. Stays were longer in autistic adults (8.5 days) and adults with ID (9.8 days) than in adults with ADHD (6.1 days) or another mental health condition (6.7 days). Cost per day was highest in adults with another mental health condition ($5390 compared to $4077 for autistic adults). Across all groups, most stays were based on emergent issues that were not elective or for scheduled procedures, and admission through the emergency department was seen in about two-thirds of adults in all four groups. Medicaid (40.6 %) and Medicare (24.7 %) were the primary expected payer for the majority of stays for autistic adults. The use of Medicaid as the primary payer was similar across groups. However, Medicare was more common in adults with ID (42.1 %) but less common in adults with ADHD (14.7 %) than autistic adults. About 1 in 12 (12.6 %) autistic adults had an injury code in their record upon admission, this was similar to adults in the other groups.

The most common reasons for mental MHH varied by group (Table 2). Schizophrenia was the most common reason for MHH in autistic adults and adults with ID: 30.4 % of all MHH in autistic adults and 51.0 % of all MHH in adults with IDD had schizophrenia as the principal diagnosis. In adults with ADHD and adults with other mental health conditions, depression was the most common reason for MHH (27.3 % for ADHD and 26.3 % for other conditions). Trauma- and stressor-related disorders and disruptive, impulse-control and conduct disorders were in the top five most common reasons for MHH in autistic adults and adults with ID. These conditions did not appear in the top five for adults with ADHD and adults with other mental health conditions. Alcohol-related disorders were the second most common reason for MHH in adults with other mental health conditions but did not make the top five in other adults.

### 30-day all-cause readmission

**Fig. 2.**
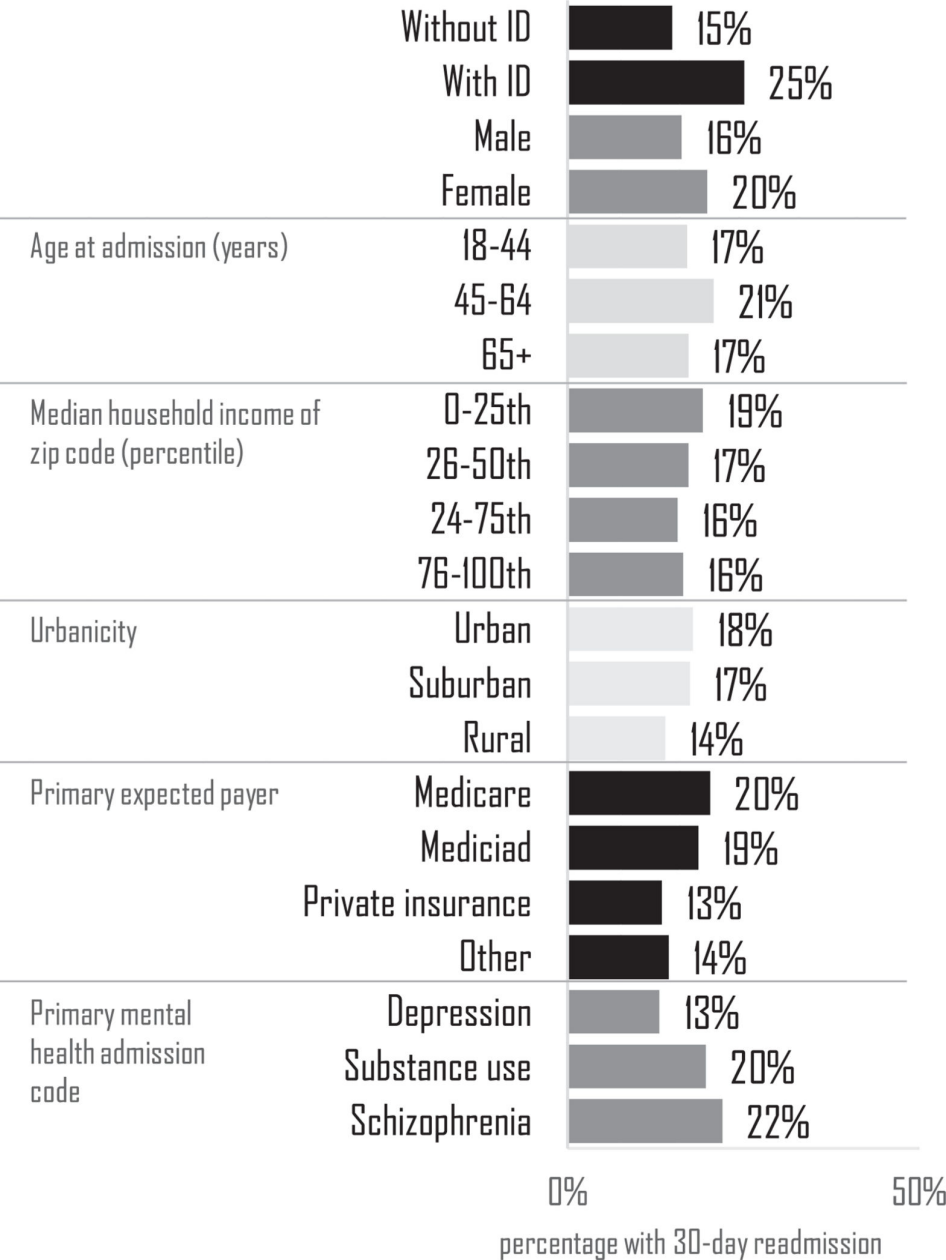
Readmission prevalence by key characteristics in autistic adults.

Of all the MHH index events in autistic adults, 17 % had a 30-day all-cause readmission, as did 25 % of adults with ID, 17 % of adults with ADHD, and 14 % of adults with other mental health diagnoses (Fig. 1). Further examination by characteristics in autistic adults (Fig. 2) revealed that readmissions were more common in autistic adults with ID (25 %) than in autistic adults without ID (15 %). This is commensurate with the finding that the readmission rate is 25 % in adults with ID without autism. Readmission was also slightly more common in female autistic adults (20 %) than male autistic adults (16 %). Readmission prevalence also varied by primary expected payer in autistic adults: readmission was more common among those with Medicare (20 %) or Medicaid (19 %) than those with private insurance (13 %) or another primary expected payer (14 %). Readmission prevalence also varied based on the reason for the index MHH, as 22 % those who had a primary mental health admission code of schizophrenia had a readmission, compared to 13 % with a primary code of depression.

The most common reasons for hospital readmission varied somewhat by group (Table 2). Schizophrenia was the most common reason for readmission in autistic adults (36.4 %), adults with ID (46.8 %), and adults with other mental health conditions (22.3 %) (it was number three in adults with ADHD (17.2 %)). In autistic adults, neurodevelopmental disorders were among the top five reasons for readmission (4.1 %).

The odds of 30-day all cause readmission was higher for autistic adults (OR 1.49, 95 % CI 1.40, 1.60), adults with ID (OR 1.82, 95 % CI 1.73, 1.92), and adults with ADHD (OR 1.46, 95 % CI 1.41, 1.52) than adults with mental health conditions (Table 3). Admission for schizophrenia at the index event was associated with increased odds of readmission (OR 1.37, 95 % CI 1.32, 1.42), while admission for depression was associated with a decreased odds of readmission (OR 0.86, 95 % CI 0.84, 0.88). Having an injury present during the index stay was also associated with reduced odds of readmission (OR 0.73, 95 % CI 0.71, 0.76).

We repeated this regression within autistic adults only, and there were not many differences in what was associated with readmission within the group. However, we did find that in autistic adults, females had greater odds of readmission (OR 1.30, 95 % CI 1.09, 1.55), but they had reduced odds of readmission in the model with all MHHs included (and autism as a covariate).

## Discussion

Mental health hospitalization (MHH) is a common reason for hospitalization in autistic adults. In this study, 36 % of all admissions for autistic adults in 2019 were for MHH. This was similar to adults with ADHD (41 %), but higher than adults with ID (23 %) and adults with other mental health conditions (12 %), suggesting a heightened need for accessible mental health care in autistic adults and adults with ADHD. The presentation of crisis mental health concerns in autistic adults and in adults with ID may be more complex than peers, as autistic adults and adults with ID have the longest stays in this study. The reasons for readmission also suggest different origins or presentations of mental health concerns in autistic adults and adults with ID. Schizophrenia was a much more common reason for index MHH in autistic adult and adults with IDD than adults with ADHD and adults with other mental health conditions, where depression and alcohol-related disorders were more common. Schizophrenia is more commonly diagnosed in autistic adults than in adults without autism, which may explain some of the difference in rates seen in this study (Chien et al., 2021; Croen et al., 2015). However, it may also be related to the difficulty of differential diagnosis in settings without expertise in autism. For example, some autism behaviors or autism-associated neurodevelopmental conditions may be interpreted as symptoms of schizophrenia in non-autistic adults making differential diagnosis is difficult (Chien et al., 2021; Gadow & Drabick, 2012). Trauma- and stressor-related disorders were also common reasons for MHH in autistic adults and adults with ID, which supports a growing body of work stressing a need for trauma-informed care in autistic adults (Faccini & Allely, 2021; Houck & Dracobly, 2023).

### Implications

The discontinuity of the mental health care system is a barrier to continuous quality care across the continuum of primary to crisis care. This likely contributes to the increased rate of both MHH and readmission seen in certain groups in this study. Another contributor to the increased rates of MHH seen in autistic adults and adults with ADHD is the increased rates of co-occurring mental health conditions in these groups (Kerns et al., 2020; Pehlivanidis et al., 2020). Increased MHH and subsequent readmission are compounded in adults with disabilities, particularly those with intellectual and neurodevelopmental disorders (like autism and ADHD), given additional barriers to appropriate care.

The highest rates of readmission were seen in adults with ID (including those with co-occurring autism). This suggests that barriers to quality outpatient care may be greatest in this group, including lack of services and practitioners to support this group, poor-quality or inappropriate services to meet needs, and deficits in practitioner and care navigator knowledge (Whittle et al., 2018). Healthcare practitioners, particularly adult health and mental health care providers, report feeling inadequately prepared to care for autistic patients and patients with ID, sometimes misattributing mental health symptoms to their intellectual disability (Ee et al., 2022; Zerbo et al., 2015), which is a barrier to appropriate care (Maddox et al., 2020). There are also few treatment modalities that are adapted for autistic adults (Kerns et al., 2016). Another possible explanation for the high rates of readmission in adults with ID lies in the disconnect between the state developmental disability (DD) and mental health service systems. Adults with ID are historically the group with the most direct access to the DD service system as many states use ID diagnosis and IQ as eligibility criteria for services (Barth et al., 2020; Hall-Lande et al., 2011). Adults with ID, whether or not they are also autistic, are likely to receive support through DD systems, where most providers do not have expertise in mental health care (Maddox et al., 2020; Maddox & Gaus, 2019). Even if adults with ID present with mental health systems, they are likely referred to the DD service system. Adults with ID may face unique systemic and interpersonal barriers to connecting to mental health care and mental health concerns may not be addressed as they emerge (Ee et al., 2022).

Several other characteristics were both elevated in certain groups in the index MHH and were important considerations for readmission. Medicaid was the primary expected payer for about 40 % of index MHH in autistic adults, adults with ID, and adults and ADHD, and Medicare was similarly common in adults with ID. Medicare was less common in autistic adults and adults with other mental health conditions, and even less common in adults with ADHD. In adjusted analyses, having Medicaid or Medicare as a primary expected payer were among the strongest associations with all-cause readmission. Prior studies have highlighted people with public health insurance have higher rates of readmission following a MHH (Rieke et al., 2016; Smith et al., 2015). This may be due to differing profiles of patients who are eligible for Medicaid (income or disability eligibility criteria) or Medicare (age and disability eligibility criteria), the care that is covered by these programs (Medicaid beneficiaries may be more likely to receive care in emergency settings than privately insured individuals), and differential enrollment in these programs (more women are enrolled in Medicaid than men) (Allen et al., 2021; Kaiser Family Foundation, 2025). Changes targeted at Medicaid and Medicare behavioral health coverage may then be especially impactful in reducing readmission. Median household income of the patient’s zip code also varied by group, with almost half of adults with ID living in neighborhoods with a median zip code below the 25th percentile, and in multivariable analysis, living in these zip codes was associated with an increase in the odds of readmission. Prior studies have also found an increased risk of readmission for people with lower incomes (Bui & Moriuchi, 2021), suggesting a need for outpatient care that is accessible and affordable. Finally, rurality was an important consideration for readmission, where rural patients had reduced odds of readmission. This has been seen in other studies of hospitalization and readmission (Wen et al., 2024) and may be due to recent findings of higher quality inpatient psychiatric care in rural versus urban locations (Hung et al., 2023).

Finally, within autistic adults, our findings highlighted two groups in need of additional attention, including females and people with schizophrenia. In autistic adults, females have greater rates and odds of readmission than autistic males. This is the opposite of what is seen in non-autistic populations, both in our study and in previous research (Zhou et al., 2023). This may be suggestive of inadequate inpatient and outpatient follow-up care for autistic women than men. Much research and clinical attention has focused on autism in males, with only recent attention paid to the differences in presentation and support needs for autistic women (Gould, 2017). Women may also face additional risk factors for poor mental health, including increased camouflaging and masking behaviors (Beck et al., 2020). This difference from population occurrence highlights the need for further attention on the population of autistic women generally, and their mental health needs in particular.

Schizophrenia was a risk factor for readmission in autistic adults, as it was in the other groups in this study and is in prior literature (Zhou et al., 2023). Co-occurring schizophrenia may result in greater barriers to services for autistic people because the siloing of service systems that support people with disabilities makes receipt of coordinated, continuous services difficult (Son et al., 2019). Adults with autism, especially those with ID, may receive services and support through state developmental disability (DD) services, while adults with schizophrenia may seek support through mental health systems and providers. A person with both conditions may have difficulty finding providers that feel confident managing both (Constantino et al., 2020; Son et al., 2019). Unaddressed schizophrenia may result in aggressive behavior that impacts social support, residential and day placements, care received, and social and professional relationships, resulting in long lasting negative impacts (Constantino et al., 2020). Adequate outpatient care for this group is imperative to reducing unwanted outcomes, such as readmission as examined in this study.

### Limitations

There are several data and study limitations to highlight. The National Readmission Database (NRD) is a record of hospital stays experienced in 2019. The rate of admission for mental health conditions captures the percentage of all hospital stays that were for mental health conditions, and is not a prevalence of MHH in autistic adults. The classification of a hospitalization as a MHH relies on coding behavior of admitting physicians or staff and may not accurately capture clinical presentation. Further, there may be differences in coding behavior based on autism diagnosis. This difference is not known to be true, but studies do suggested that misdiagnosis and differentiation of autism and other clinical symptoms may be prevalent in autistic adults (Kentrou et al., 2024). However, presentation of symptoms that escalates to the need for hospital admissions is a notable event, whether or not the diagnosis is clinically correct. The NRD does not contain detailed information about patient characteristics or medication use, which could be pertinent information about care occurring during an MHH. Similarly, NRD does not contain information or care occurring after discharge or in outpatient settings, which would be illuminating in the examination of readmissions.

## Conclusions

The complexity of mental health conditions and their care in autistic adults underscores the urgent need for accessible and tailored mental health care services. Our findings reveal higher readmission rates in autistic adults, particularly among those with intellectual disabilities (ID), emphasizing the importance of addressing the unique support needs of this population. The difference in readmission rates between autistic males and females may highlight the necessity for better identification and support of mental health in autistic females. Additionally, the intersectionality of autism and schizophrenia further complicates access to continuous care, emphasizing the imperative for integrated service systems to bridge existing gaps. Addressing these challenges will require collaborative efforts across healthcare sectors to create comprehensive, inclusive, and person-centered approaches to mental health care delivery for autistic adults. Next steps should focus on understanding the adequacy of discharge follow-up services to ameliorate disparities in readmission. Other system-level interventions to decrease rates of mental health crisis care and improve outpatient mental health care are needed.

## Figures and Tables

**Table 1:** Demographic and stay characteristics of the index mental health hospitalization.

	Autism		Intellectual disability (ID)		Attention deficit hyperactivity disorder (ADHD)		Other mental health conditions	
Demographic characteristics	%	SE	%	SE	%	SE	%	SE

Age at admission (mean)	28.5	0.2	38.6	0.3	33.4	0.1	43.2	0.2
Female	28.7	0.6	46.3	0.6	42.9	0.4	45.0	0.3
Median household income for patient’s ZIP Code								
0 −25th percentile	29.1	1.3	46.5	1.4	27.7	1.0	36.5	0.9
26th to 50th percentile	28.4	0.9	28.0	0.9	27.5	0.8	26.5	0.6
51st to 75th percentile	24.6	0.9	17.4	0.8	25.3	0.7	21.9	0.5
76th to 100th percentile	18.0	1.0	8.1	0.6	19.5	1.0	15.1	0.7
Urbanicity								
Urban	45.7	2.0	49.2	2.0	44.1	1.9	48.7	1.6
Suburban	40.3	1.9	31.7	1.7	41.9	1.8	36.3	1.5
Rural	13.9	1.0	19.1	1.2	14.0	0.9	15.0	0.7
**Stay characteristics**								
Length of stay (mean)	8.5	0.3	9.8	0.3	6.1	0.1	6.7	0.1
Cost per day (mean)	$4077	102	$3784	97	$4522	107	$5390	117
Emergency department use	63.4	1.8	65.3	1.8	65.2	1.6	67.2	1.3
Elective admission	7.8	0.7	9.5	0.9	9.3	0.8	9.3	0.6
Primary expected payer								
Medicare	24.7	0.6	42.1	0.9	14.7	0.3	23.7	0.4
Medicaid	40.6	1.0	44.3	1.2	41.3	1.0	36.0	0.8
Private insurance	27.3	1.1	9.1	1.2	30.2	0.9	24.4	0.7
Other	7.3	0.5	4.5	0.4	13.8	0.7	16.0	0.7
Diagnoses recorded (mean)	9.7	0.1	10.9	0.2	11.2	0.1	10.4	0.1
Depression primary code	25.4	0.8	16.1	0.6	27.3	0.7	26.3	0.6
Substance use primary code	3.9	0.3	3.8	0.3	21.5	1.0	28.2	0.8
Schizophrenia primary code	30.4	0.9	51.0	1.0	14.2	0.4	19.6	0.5
Injury ICD10 code in record	12.6	0.5	9.1	0.3	15.8	0.4	14.2	0.2

**Table 2:** Most common principal diagnoses for index admission and readmission by group.

*Most common principal diagnoses for the index mental health hospitalization*								
	Autism		Intellectual disability (ID)		Attention deficit hyperactivity disorder (ADHD)		Other mental health conditions	
	*Condition*	*%*	*Condition*	*%*	*Condition*	*%*	*Condition*	*%*

1st	Schizophrenia	30.4	Schizophrenia	51.0	Depression	27.3	Depression	26.3
2nd	Depression	25.4	Depression	16.1	Bipolar	22.3	Alcohol	19.7
3rd	Bipolar	19.9	Bipolar	14.3	Schizophrenia	14.2	Schizophrenia	19.6
4th	Trauma	5.0	Trauma	4.9	Alcohol	11.2	Bipolar	13.6
5th	Disruptive	4.4	Disruptive	3.3	Suicide	4.5	Suicide	4.3
*Most common principal diagnoses for the readmission*								
1st	Schizophrenia	36.4	Schizophrenia	46.8	Bipolar	20.3	Schizophrenia	22.3
2nd	Bipolar	17.0	Depression	12.1	Depression	17.3	Depression	15.1
3rd	Depression	16.6	Bipolar	11.9	Schizophrenia	17.2	Alcohol	15.2
4th	Neurodevelop	4.1	Trauma	3.1	Alcohol	12.6	Bipolar	11.7
5th	Disruptive	3.5	Disruptive	1.9	Opioid	2.9	Septicemia	2.0

Diagnoses based on Clinical Classifications Software Refined (CCSR) codes and are defined as follows: Schizophrenia spectrum and other psychotic disorders (MBD001); Depression and depressive disorders (MBD002); Bipolar and related disorders (MBD003); trauma- and stressor-related disorders (MBD007); Disruptive, impulse-control and conduct disorders (MBD008); Suicidal ideation/attempt/intentional self-harm (MBD012); Neurodevelopmental disorders (MBD014); Alcohol-related disorders (MBD017); Opioid-related disorders (MBD018); and septicemia (INF002).

**Table 3:** Multivariable logistic regression estimating odds of readmissions by index characteristics.

	All-cause readmission within 30 days		All-cause readmission within 30 days, autism only	
	OR	95 % CI	OR	95 % CI

**Condition**				
Autism	1.49	[1.40,1.60]		
ID	1.82	[1.73,1.92]		
ADHD	1.46	[1.41,1.52]		
Other mental health	reference			
Autism with ID			1.96	[1.62, 2.37]
Autism without ID			reference	
**Inpatient demographic characteristics**				
Age at admission	1.01	[1.01,1.01]	1.00	[0.99,1.01]
Female	0.83	[0.82,0.85]	1.27	[1.06,1.52]
Median household income for ZIP				
0 —25th percentile	1.16	[1.10,1.21]	1.00	[0.78,1.27]
26th to 50th percentile	1.07	[1.03,1.11]	0.96	[0.76,1.22]
51st to 75th percentile	1.02	[1.00,1.06]	0.88	[0.69,1.13]
76th + percentile	reference		reference	
Urbanicity				
Urban	reference		reference	
Suburban	0.99	[0.95,1.03]	1.00	[0.84,1.20]
Rural	0.80	[0.76,0.84]	**0.64**	**[0.49,0.85]**
**Stay characteristics**				
Mean length of stay	1.00	[0.99,1.00]	1.00	[0.99,1.01]
Mean cost per day	1.00	[1.00,1.00]	1.00	[1.00,1.00]
Emergency department use	1.13	[1.08,1.18]	1.26	[1.05,1.50]
Elective admission	0.89	[0.84,0.94]	1.10	[0.73,1.64]
Primary expected payer				
Medicare	1.56	[1.50,1.62]	1.35	[1.04,1.75]
Medicaid	1.56	[1.50,1.63]	1.22	[0.99,1.50]
Private insurance	reference		reference	
Other	1.22	[1.16,1.29]	0.94	[0.66,1.34]
Number diagnoses recorded	1.03	[1.02,1.03]	1.03	[1.01,1.04]
Depression as primary code	0.86	[0.84,0.88]	0.93	[0.76,1.15]
Substance use as primary code	1.11	[1.07,1.16]	1.59	[1.09,2.32]
Schizophrenia as primary code	1.37	[1.32,1.42]	1.34	[1.10,1.64]
Injury ICD10 code in stay record	0.73	[0.71,0.76]	0.82	[0.64,1.05]

## Data Availability

The authors do not have permission to share data.
